# The complete mitochondrial genome of *Xylotrechus namanganensis* (Coleoptera: Cerambycidae)

**DOI:** 10.1080/23802359.2022.2094293

**Published:** 2022-07-12

**Authors:** Zhengpeng Yan, Laibin Zhang, Haibing Xiao, Minglu Yang

**Affiliations:** a College of Agriculture, Tarim University, Alar, P. R. China; b Key Laboratory of Integrated Pest Management on Crops in Southern Xinjiang, Xinjiang Production and Construction Corps, Alar, P. R. China

**Keywords:** *Xylotrechus namanganensis*, *Turanoclytus namanganensis*, mitochondrial genome, Namangan longhorn beetle

## Abstract

The complete mitochondrial genome of *Xylotrechus namanganensis* was sequenced. The genome size was 15,565 bp, which consists of 13 protein-encoding genes, 22 tRNA-encoding genes, 2 rRNA-encoding genes and 1D-loop control region. The base composition of mitogenome was biased toward A + T content, of which was 73.21%. The phylogenetic tree based on complete mitogenome sequences revealed that *T. namanganensis* had a closer relationship with *X. grayii*.

*Xylotrechus namanganensis* (Heyden 1885, homotypic synonym of *Turanoclytus namanganensis*) is named as Namangan longhorn beetle. It is a borer pest to many broad-leaved tree species in Xinjiang, including *Ulmus spp*., *Populus alba*, *Populus nigra*, *Platanus* × *hispanica*, *Salix spp.*, *Malus domestica*, *Morus nigra*, *Elaeagnus angustifolia*, *Celtis australis*, *Juglans regia*, *Alnus glutinosa*, *Populus* spp., *Prunus* spp. (Wang 2001; EPPO, European and Mediterranean Plant Protection Organization 2005; Li 2018; Hănceanu et al. 2021). Adults of this pest only take in honey in flowers or juice of the trees, and they do not directly destroy the plants. Whereas, its eggs, larvae and pupae live within trunks, delay sprouting, advance leaf shedding, and halter growth of the infected trees (Wang et al. 1999). The larva can be found all the year around, and the adult and egg can be found from the middle of April to the late of July. On the basis of recognizing eclosion cavity of elm trees, spatial distribution of *T. namanganensis* on the trunk is an assembly distribution model (Wang et al. 2000). In this study, we sequenced the complete mitochondrial genome of *T. namanganensis* to further investigate its phylogenetic relationships of Cerambycidae.

The larva samples of *T. namanganensis* were collected from Kunyu, Xinjiang province, China (37°22′05ʺN, 79°15′44ʺE), and the specimen was deposited at the insect specimen room of Tarim University (Yan Zhenpeng, 1733047107@qq.com) under the voucher number KY2021061102. The total genomic DNA was followed by library preps and pair-end sequenced (2 × 150 bp) with Illumina HiSeq4000 platform. Approximately 45,620 Mb of raw data and 45,320 Mb of clean data were obtained, and de novo assembly, annotation and visualization were conducted using the Mitoz novo software (v2.4) according to Meng et al. (2019).

The mitogenome of *T. namanganensis* was 15,564 bp (NCBI accession number OK189517), containing 13 protein-coding genes (PCGs), 22 transfer RNA genes (tRNAs), 2 ribosomal RNA (12S rRNA and 16S rRNA) genes, and a non-coding control region (D-loop). The mitogenome base composition of *T. namanganensis* was biased toward A + T content, of which was 73.21% (40.14% A, 33.07% T, 16.22% C, 10.57% G). The 13 identified PCGs varied from 157 to 1,741 bp in length. The lengths of the 23 tRNA genes ranged from 64 to 71 bp, and all of the tRNA genes contained typical secondary structure. The 12S rRNA gene was located between the genes of tRNA-Val and tRNA-Ile, and was 780 bp in length, while the 16S rRNA gene was located between tRNA-Leu and tRNA-Val, with a length of 1,331 bp.

A neighbor-joining phylogenetic tree of *T. namanganensis* with ten other closely related species was constructed based on the complete mitochondrial genomes using the MEGA-X method (Kumar et al. 2018) (Figure 1). The result suggested that *T. namanganensis* is closely related to *X. grayii*, and both of them belong to the genus of *Turanoclytus*.

**Figure 1.**
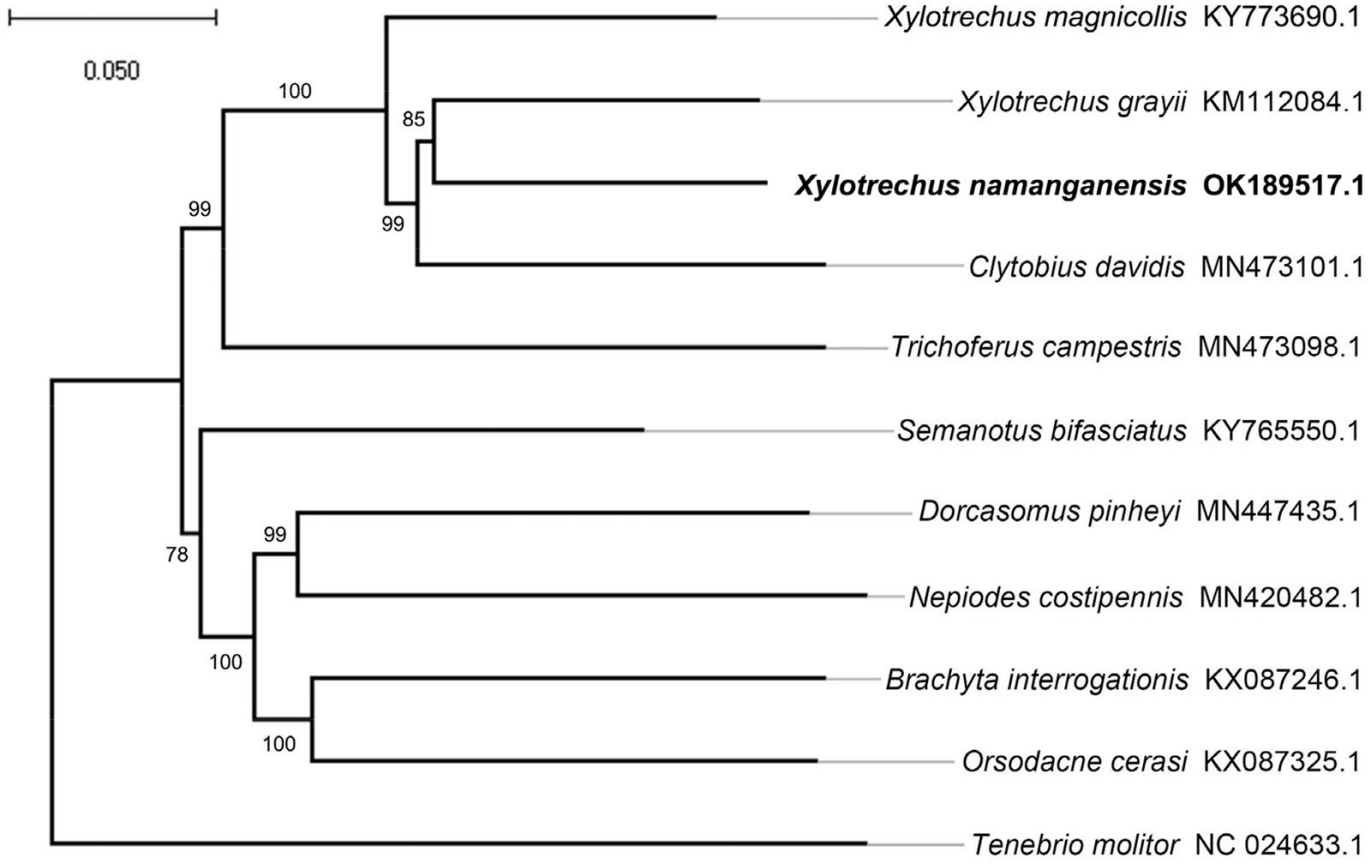
Neighbor-joining phylogenetic tree of *Xylotrechus namanganensis* and ten other closely related species based on the complete mitochondrial genomes. The nodal numbers indicated the bootstrap values obtained with 1000 replicates. *Tenebrio molitor* was selected as an outgroup.

## Data Availability

The genome sequence data that support the findings of this study are openly available in GenBank of NCBI at (https://www.ncbi.nlm.nih.gov/) under the accession no. OK189517.1. The associated BioProject, SRA, and Bio-Sample numbers are PRJNA760792, SRR16700842, and SAMN21223651 respectively.
